# *StTCP15* regulates potato tuber sprouting by modulating the dynamic balance between abscisic acid and gibberellic acid

**DOI:** 10.3389/fpls.2022.1009552

**Published:** 2022-09-16

**Authors:** Kaitong Wang, Ning Zhang, Xue Fu, Huanhuan Zhang, Shengyan Liu, Xue Pu, Xiao Wang, Huaijun Si

**Affiliations:** ^1^State Key Laboratory of Aridland Crop Science, Gansu Agricultural University, Lanzhou, China; ^2^College of Agronomy, Gansu Agricultural University, Lanzhou, China; ^3^College of Life Science and Technology, Gansu Agricultural University, Lanzhou, China

**Keywords:** potato, *StTCP15*, dormancy, sprouting, ABA, GA_3_

## Abstract

The major stages of the potato life cycle are tuber dormancy and sprouting, however, there is still known very little of the mechanisms that control these processes. TCP (Theosinte branch I, Cycloidea, proliferationcell factors 1 and 2) transcription factors play a key role in plant growth and dormancy related developmental processes. Previous researches demonstrated that TCP transcription factor *StTCP15* had a function in the promotion of dormancy. To elucidate the function of *StTCP15* gene, it was cloned from potato cultivar “Desiree,” which encodes a polypeptide consisting of 414 amino acids and is mainly found in the nucleus. The potato tubers of *StTCP15* overexpression lines sprouted in advance, while the potato tubers of *StTCP15* down-regulated expression lines showed delayed sprouting. In addition, it was also found that overexpression lines of *StTCP15* extremely significantly reduced the ratio of abscisic acid (ABA)/gibberellic acid (GA_3_), while the superoxide dismutase activity decreased, and the activity of peroxidase and catalase increased compared with the wild type. The opposite result was found in the down-regulated expression lines of *StTCP15* gene. Three interacting proteins, StSnRK1, StF-Box and StGID1, were screened by Yeast two-hybrid, and verified by Bimolecular Fluorescence Complementation and Split-luciferase, indicating that *StTCP15* could affect ABA and GA_3_ signaling pathways to regulate potato tuber dormancy and sprouting. Together, these results demonstrated that *StTCP15* regulated potato tuber dormancy and sprouting by affecting the dynamic balance between ABA and GA_3_. The result could provide some information on the molecular mechanism of *StTCP15* regulating potato tuber dormancy and sprouting.

## Introduction

Potato (*Solanum tuberosum* L.) is a vital meal crop grown in many countries and regions around the world. Potato tuber dormancy has a great impact on its cultivation, processing and storage. People have different demands on potatoes and different requirements on the length of the dormancy period, which makes the length of the resting period of potatoes of great biological significance (Deligios et al., 2020; Gong et al., 2021). Numerous studies have shown that seed dormancy and germination are controlled by multiple hormones and environmental signals, among which plant hormones abscisic acid (ABA) and gibberellic acid (GA_3_) are the major endogenous hormones antagonizing seed dormancy and germination (Gubler et al., 2005; Finkelstein et al., 2008). It has been shown that seed germination is determined by the homeostasis of endogenous ABA and GA_3_ levels in seeds, germination is usually accompanied by decreased ABA levels and increased GA_3_ levels. Therefore, lower ABA levels and improve the level of GA_3_ is necessary for seed dormancy release and then sprouting (Yang et al., 2020), but the balance of how to regulate it is unclear.

TCP (Teosinte branched I, Cycloidea, Proliferating Cell Factors 1 and 2) is a group of plant-specific transcription factors that contain a basic helix-loop-helix (bHLH) motif consisting of 59 amino acids, which is responsible for DNA binding and protein-protein interactions (Cubas et al., 1999; Heim et al., 2003). From the similarity of amino acid sequences in the TCP domain, TCP proteins can be divided into two categories: class I (PCF or TCP-P) and class II (TCP-c, CYC/TB1/CIN-like). Class I proteins participate in the regulation of growth patterns by controlling cell proliferation and hormone signaling pathways (Camoirano et al., 2020), but class II protein members inhibit these processes (Herve et al., 2009; Li et al., 2019). In *Arabidopsis thaliana*, class I family members *AtTCP14* and *AtTCP15* participate in the gibberellin signaling pathway to promote embryonic development, thereby regulating seed dormancy in advance (Tatematsu et al., 2008). AtTCP14 indirectly inhibits ABA biosynthesis gene *ABA1* and stress-related genes regulated by ABA through interaction with DOF6, resulting in delayed germination (Rueda-Romero et al., 2012). Resentini et al. (2021) found that AtTCP15 interacted with DELLA protein GAI and RGL2 to mediate GA to promote *A. thaliana* seed germination. *AtTCP20* has also been reported to play a role in different developmental stages, jasmonic acid synthesis and leaf senescence in *A. thaliana* (Guan et al., 2014). *GhTCP19* transcription factor affects the regulation of dormancy release in gladiolus corms by repressing *GhNCED* (ABA biosynthesis gene) expression (Wu et al., 2019). Guo et al. (2018) showed that peach *PpTCP.A2* could regulate fruit ripening process by affecting the expression of ET biosynthesis 1-aminocyclopropane-1-car-boxyla synthase (*PpACS1*) gene. Overexpression of *OsTCP19* in *Arabidopsis* leads to up-regulation of *IAA3*, *ABI3* and *ABI4* and down-regulation of *LOX2*, resulting in developmental abnormalities (Mukhopadhyay and Tyagi, 2015). In class II family members, AtTCP3 indirectly activates transcription of DELLA protein-coding gene *GAI*, thereby regulating gibberellin activity (Nicolas and Cubas, 2016). *SlTCP26* negatively regulates auxin signal to reduce apical dominance and inhibits abscisic acid signal to release lateral bud dormancy and promote lateral branch development (Wei et al., 2021). The TCP family gene *CsBRC1* directly inhibited the expression of *CsPIN3*, promoted the accumulation of IAA in axillary buds, and inhibited the germination of axillary buds (Shen et al., 2019). *VcTCP18* negatively regulated the dormancy release of flower buds, and the seed germination rate of transgenic *A. thaliana* was lower than that of the wild-type. In transgenic plants, *VcTCP18* showed later flowering and less rose inflorescence and main stem (Li Y. et al., 2021).

In our previous studies, we found that the expression level of potato *StTCP15* (GenBank No. Xm_006364123.2) was continuously up-regulated during tuber dormancy release Wang et al. (2019), suggesting that this gene may regulate the function of tuber dormancy release and sprouting. In this study, tissue specific expression of *StTCP15* was verified by quantitative real-time polymerase chain reaction (qRT-PCR). The expression site of StTCP15 in cells was determined by subcellular localization. The overexpression and down-regulated expression plants of *StTCP15* of potato cultivar “Desiree” were used as materials, for analysis of transgenic tuber dormancy release and sprouting response. The protein interacting with StTCP15 was screened by Y2H (Yeast two-hybrid), and the reliability of interaction was verified by BiFC (Bimolecular Fluorescence Complementation) and Split-LUC Complementation. This study provided a theoretical and applicable framework for further study of dormancy release and sprouting in potato tubers.

## Materials and methods

### Plant growth conditions and treatments

Potato (*Solanum tuberosum* L. cv. “Desiree”) containing two pieces of leaves *in vitro* plantlet successive transfer culture to Murashige-Skoog (MS) solid media containing 3 and 6% sucrose under (23 ± 1)°C, 16-h light/8-h dark cycle. After 4 weeks of culture, MS solid media containing 6% sucrose was cultured in darkness for 1 month to obtain microtubers (Zhang et al., 2005). For tissue specificity and tuber dormancy analysis, “Desiree” grown in 3% MS media for 4 weeks was transplanted into a flower pot with a diameter of 30 cm and a height of 25 cm, and placed at the optimum temperature of (23 ± 2)°C in the greenhouse of Gansu Agricultural University. After 2 weeks of culture, roots, stems and leaves were collected and quickly frozen in liquid nitrogen at a temperature below –80°C. The tubers were harvested after 12 weeks and stored at (23 ± 2)°C and under dark conditions. Samples were collected from the beginning of storage (0 day), dormancy release (30 day) and sprouting (bud length > 0.2 cm; 45 day) with a sampler with a diameter of 0.8cm (Van Ittersum et al., 1992). The samples were frozen with liquid nitrogen and stored in a –80°C refrigerator for subsequent experiments.

Tobacco (*Nicotiana Benthamian* L.) was grown and cultured in a pot of 10 cm × 10 cm filled with nutrient soil (nutrient soil: vermiculite = 1:1) under the conditions of light intensity of 2000 Lx, photoperiod of 16-h light/8-h dark and temperature of (23 ± 2)°C. The relevant experiment could be carried out after about 30 days of culture.

### Bioinformatics analysis

The *StTCP15* sequence (ID: Soltu.DM.03G03040.1) was retrieved from the potato database Spud DB^1^ and the location of chromosome exon-intron structure was searched. The divergence of exon-intron structures was analyzed using NCBI Splign. NCBI conservative Domains were used to analyze the conservative domain of StTCP15 protein. The homologous species evolutionary tree was constructed by MEGA 7.0 software. The ExPASy ProtParam^2^ tools ExPASy and ProtScale^3^ were used online they predict physicochemical properties of the protein. The NetPhos 3.1^4^ online analysis software was used to predict StTCP15 protein phosphorylation site distribution. The SPSS 13.0 software was processed to analyze the data.

### *StTCP15* expression analysis by qRT-PCR

FastKing RT Kit with gDNase (Tiangen Biotechnology, Beijing) was used for first-strand cDNA synthesis. Super Real PreMix Plus (SYBR Green; Tiangen Biotech, Beijing) was used to analyze the expression level of *StTCP15* with gene-specific primers (Supplementary Table 1). Polymerase chain reaction (PCR) solution (20 μl) contained 10 μl 2 × SuperReal PreMix Plus, 0.6 μl forward and reverse primers, 1 μL cDNA (100 ng) template, and 7.4 μl nuclease-free water. qRT-PCR was performed in the Light Cycler 96 system (Roche, Diagnostics GmbH) with the following parameters: 95°C for 15 min, followed by 40 cycles of 95°C for 10 s, 60°C for 20 s, and 72°C for 30 s. The *StEF1*α (GenBank ID: AB061263.1) gene was used as a standardized reference gene (Tang et al., 2017). The primer sequences are shown in Supplementary Table 1. All experiments were performed with three biological replicates and three technical replicates. The relative expression levels of the *StTCP15* gene in different tissues and dormancy times were calculated by 2^–ΔΔ^*^Ct^* method (Rao et al., 2013).

### Cloning and subcellular localization of StTCP15

The *StTCP15* gene sequence (ID: Soltu.DM.03G03040.1) was retrieved from potato database Spud DB (see text footnote 1). The subcellular localization vector was constructed by homologous recombination. According to the vector pCAMBIA1300-35S-EGFP sequence and *StTCP15* sequence, Using TaKaRa primer design software^5^ online design specific primers (Supplementary Table 1). The CDS sequences of *StTCP15* gene without terminator were amplified from the cDNA of potato cv. “Desiree.” The PCR product was inserted into the vector pCAMBIA1300-35S-EGFP containing *Kpn*I and *Xba*I restriction sites to obtain the recombinant plasmid pCAMBIA1300-EGFP-StTCP15. Plasmid pCAMBIA1300-EGFP-StTCP15 and empty vector pCAMBIA1300-35S-EGFP were transformed into *Agrobacterium tumefaciens* GV3101. The second to fourth leaves of tobacco were selected from the new leaves at the age of 5–6 weeks old. A small hole was punctured on the back of the tobacco with a sterile syringe needle as the injection entrance for *Agrobacterium* infection. The infection solution was injected slowly into the tobacco leaves with a syringe and the infection area was marked. *Agrobacterium* infected plants were cultured in darkness for 1 day in a 25°C incubator and then transferred to light for 1–2 days. GFP signals were detected with a confocal scanning electron microscope (CARI ZEISS, LSCM 800, Germany) at a 488 nm laser wavelength (Qi et al., 2020).

### Construction of plant expression vector

For the *StTCP15* gene sequence and the expression vector pBI121 sequence, specific primers were used to amplify the CDS sequence of *StTCP15* gene, and the gene was cloned by PCR, Primer sequences are shown in Supplementary Table 1. The PCR product was inserted into the linearized vector pBI121 containing *Bam*HI and *Sac*I restriction sites according to the homologous recombinase specification. After PCR detection, double enzyme digestion verification and sequencing, the ligated product was successfully named pBI121-StTCP15. To down-regulate *StTCP15* gene in potatoes, this experiment designed the sequence of aimRNA using the WMD3 website (Supplementary Table 1 and Supplementary Figure 1; Li et al., 2020). Standard PCR was used for cloning. PCR products to the pMD18-T vector (TaKaRa Bio, Beijing) verified PCR detection, the double enzyme digestion and sequencing, connecting the effective sequence with *Kpn*I and *Sac*I double enzyme pCPB121 vector, sequencing the right named pCPB121-amiR-StTCP15. The successfully identified recombinant plasmid vector was transformed into *Agrobacterium tumefaciens* GV3101 by repeated freezing and thawing (Hayta et al., 2018; Li S. et al., 2021).

### Potato transformation and identification

The genetic transformation of potato cultivar “Desiree” microtuber was based on the transformation method of Si et al. (2003). The microtubers were cut by a sterile blade into pieces that were 0.2–0.3 cm thick. Potato chips were transferred to *Agrobacterium* solution containing recombinant plasmids pBI121-StTCP15 and pCPB121-amiR-StTCP15 for 7–10 min. The remaining bacterial solution on potato slices was dried with sterile dry filter paper and placed in a petri dish containing solid MS for 2 days at 28°C under light protection. The co-cultured potato slices were transferred to the differentiation media and cultured at 2500 Lx at 25°C. The media was changed once a week. When the differentiated shoots grew to about 1.5 cm, they were cut and transferred to the rooting medium containing 50 mg/L kanamycin and 200 mg/L carbenicillin for rooting screening (Qi et al., 2020; Zhu et al., 2021). After about 7 days, the plants rooting on the media were preliminarily identified as transformed plants.

To identify transgenic plants, the neomycin phosphotransferase (*NPT* II) gene on the expression vector was used for PCR detection. Genomic DNA of transgenic plants was extracted by CTAB method (Li S. et al., 2021). The wild type potato cultivar “Desiree” were used as a negative control, and the constructed plant expression vector plasmid was used as a positive control. Electrophoresis detected the fragment size of 676 bp, which was transformed into pBI121-StTCP15 and pCPB121-amiR-StTCP15 transgenic potato plants, named OE-n and RNAi-n, respectively, and used for. Further identification by qRT-PCR was performed to identify successful transgenic potato plants for later experiments.

### Analysis of transgenic potato tuber

Wild-type (WT) potato plants, transgenic lines OE-n and RNAi-n were propagated and transplanted to pots to obtain potato tubers. After harvesting potato tubers, they were allowed to air dry and stored in darkness at (23 ± 2)°C. A total of 100 transgenic and WT potato tubers of the same size were selected, and the dormancy and germination of tubers were observed every 5 days since the date of harvest. The sign of dormancy removal of tubers is that at least one bud larger than 2 mm grows on tubers (Van Ittersum et al., 1992). All comparative sprouting tests were conducted using batches of tubers harvested at the same time from plants growing under the same environmental conditions.

### Measurement of superoxide dismutase, peroxidase, catalase, ABA and GA_3_ in transgenic potato tuber

The transgenic and WT potato tubers were used as materials. During the dormancy release period of tubers, tissue samples were collected from the bud eye area using a 0.5 cm diameter sampler, and the fresh plant materials were quickly placed in liquid nitrogen and stored in a low-temperature refrigerator at –80°C. Superoxide dismutase (SOD) activity was measured by NBT method (Johnson-Flanagan and Owens, 1985). The activity of peroxidase (POD) activity was determined by guaiacol method (Maehly and Chance, 1954). Catalase (CAT) activity was quantified using the method of Rajinder et al. (1982). ABA and GA_3_ contents were determined by ELISA kit (Shanghai Jianglai Biotechnology Co., Ltd., Shanghai, China) according to the instructions of the kit, as described by Zhang et al. (2009). Each experiment was set up with three biological replicates.

### Yeast two-hybrid assay

According to the vector pGBKT7 sequence and *StTCP15* gene sequence, specific primers are used to amplify the *StTCP15* sequence, and the specific primers were shown in Supplementary Table 1. The PCR product was inserted into bait vector pGBKT7 containing *Nde*I and *Not*I restriction sites, and the recombinant plasmid pGBKT7-StTCP15 was obtained. The bait vector pGBKT7-StTCP15 was co-transformed into yeast strain AH109 (Clontech) with cDNA library of potato cultivar “Zihuabai,” which was constructed by fusing cDNA with GAL4 activation domain in pGADT7-Rec vector. The inverters were screened on selective media SD/-Leu/-Trp/-His/-Ade/X-α-gal, and then the interaction of each of the two protein combinations was determined by the blue color of the colony. Further identification of blue positive clones by sequence. All assays were performed according to the protocol described in the YEASTMAKER Yeast Transformation System User Manual (Clontech).

### Bimolecular fluorescence complementarity assay

In order to verify the reliability of the Y2H, this research will PCR amplification without termination codon *StTCP15* coding sequence containing *Bam*HI and *Sam* I restriction sites in the pSPYCE-35S vector; The full-length coding sequences of *StSnRK1*, *StF-box* and *StGID1* without termination codon were inserted into pSPYNE-35S containing *Bam*HI and *Sam* I restriction sites. pSPYCE-StTCP15 and pSPYNE-35S empty vectors were used as negative controls for BiFC analysis. The constructed BiFC vector was transformed into *Agrobacterium tumefaciens* GV3101, and the bacterial solution was mixed 1:1 with a sterile syringe. The injection and infection methods were the same as the subcellular localization method. After 48–72 h, the yellow fluorescent protein (YFP) signal was observed by confocal scanning electron microscope (CARI ZEISS, LSCM 800, Germany; Ma et al., 2021).

### Split-LUC complementation assay

The CDS of *StTCP15* was cloned into the *Kpn*I and *Xba*I sites of the pCAMBIA1300-cLUC vector, and the *StSnRK1*, *StF-box*, and *StGID1* without terminator CDS were ligated to the *Sac*I and *Sal*I sites of the pCAMBIA1300-nLUC vector. Specific primers were shown in Supplementary Table 1. The recombinant plasmid was transformed into *Agrobacterium tumefaciens* strain GV3101. As described by Chen et al., pCAMBIA1300-cLUC-StTCP15 and pCAMBIA1300-nLUC-StSnRK1/StF-box/StGID1 were mixed in equal volumes, and the injection and infection methods were the same as the subcellular localization methods. pCAMBIA1300-cLUC and pCAMBIA1330-nLUC served as blank controls. *Nicotiana benthamiana* leaves were sprayed with D-luciferin potassium salt (Solarbio, Beijing, China), and the fluorescence was detected by a plant live imaging system (BLT PlantView100, Guangzhou Biolight Biotechnology Co., Ltd., Guangzhou, China; Chen et al., 2022).

## Results

### Bioinformatics and expression analysis of *StTCP15* gene

The potato *StTCP15* gene bioinformatics analysis results showed that *StTCP15* gene is 1981 bp in length, which contains 1245 bp CDS sequence, encoding a polypeptide of 414 amino acids. By analyzing NCBI Splign, *StTCP15* genes were found to contain one intron and two exons, which was a discontinuous gene (Supplementary Figure 2A). The gene was located on chromosome 3 (PGSC gene ID: Soltu.DM.03G03040.1; Chromosome location: chr03:54904362-54906342). According to NCBI conservative domain analysis, it was found that StTCP15 contains TCP domain (95-166AA; Supplementary Figure 2B). The total molecular weight of the protein was 44.26 KD and PI 7.06, indicating that the protein was neutral. The grand average of hydropathy is –0.713, which is a hydrophilic protein. There were 63 phosphorylation sites with a total score of 0.5–1, accounting for 15.22% (Supplementary Figure 2C), which provided conditions for the function of transcription factors (Yang et al., 2021).

**FIGURE 1:**
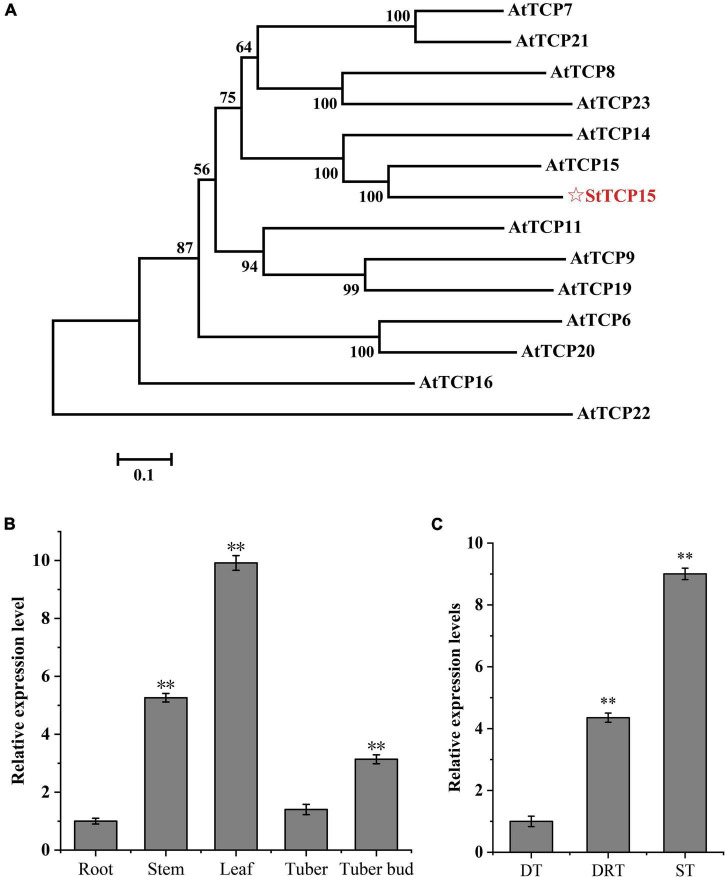
Analysis of potato StTCP15. **(A)** The phylogenetic tree of PCF class and StTCP15 in *Arabidopsis thaliana*. **(B)** The relative expression level of *StTCP15* in different organs of potato. **(C)** The relative expression levels of *StTCP15* genes during release of tuber dormancy. DT: Dormant tuber (0 day); DRT: Dormant release tuber (30 day); ST: Sprouting tuber (45 day). Relative expression levels, determined by qRT-PCR, relative to the expression of the *StEF1*α gene, expressed as 2^–ΔΔCt^. Each column represents the mean values ± SE (*n* = 3; **P < 0.01).

The phylogenetic relationship between PCF class and StTCP15 protein in *A. thaliana* was analyzed, and it was found that StTCP15 and AtTCP15/14 had the closest homology (Figure 1A). Studies have shown that AtTCP15/14 has a regulatory role in the process of seed dormancy release and germination (Tatematsu et al., 2008), and StTCP15 may regulate the dormancy release and sprouting of potato tubers.

Based on the qRT-PCR results, the *StTCP15* gene was expressed in all potato tissues with significant difference (*P* < 0.01). The relative expression level in leaves was 9.91 times that in roots with the lowest relative expression level, and the expression level in stems, tubers and buds was 5.26, 1.40, and 3.14 times that in roots, respectively (Figure 1B). The expression of *StTCP15* gene increased during dormancy release, and the difference between dormancy and dormancy release was extremely significant (*P* < 0.01), dormancy release and sprouting were about 4.35 and 9.00 times that in dormancy, respectively (Figure 1C).

### Subcellular localization of StTCP15-EGFP fusion protein

**FIGURE 2:**
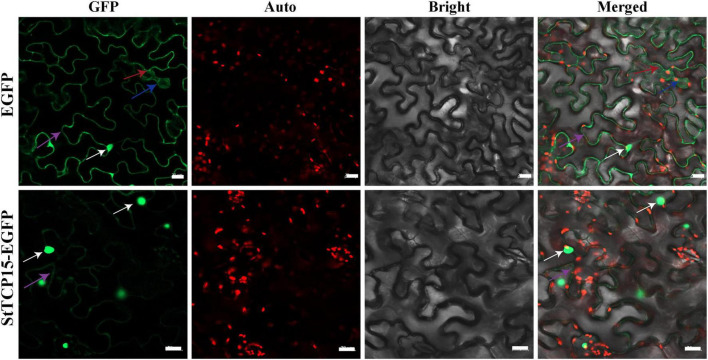
Subcellular localization of StTCP15 protein in *Nicotiana benthamiana* leaf cells. The EGFP and StTCP15-EGFP fusion protein transiently expressed in tobacco. The white arrows indicate the nucleus, blue arrows indicate cytoplasm, purple arrows indicate cytoplasmic membrane, and red arrows indicate chloroplasts. GFP: EGFP fluorescence signal in the dark field; Auto: Autofluorescence of chlorophyll; Bright: Cell morphology under bright field; Merged: Combination field. The scale bale represents 20 μm.

StTCP15-EGFP fusion protein was injected into tobacco by *Agrobacterium*-mediated transformation and expressed in leaf cells of tobacco. The expression of the fusion protein was observed under a laser confocal scanning microscope (pCAMBIA1300-35S-EGFP empty vector as negative control). The results showed that StTCP15-EGFP fusion protein had strong GFP fluorescent signal mainly in the nucleus and a weak GFP fluorescent signal in the cell membrane. However, the control GFP fluorescent signal was located in the whole tobacco cell, including the cell membrane, cytoplasm, and nucleus, without specific compartmentalization (Figure 2).

### Genetic transformation of *StTCP15* and identification of transgenic potato plants

**FIGURE 3:**
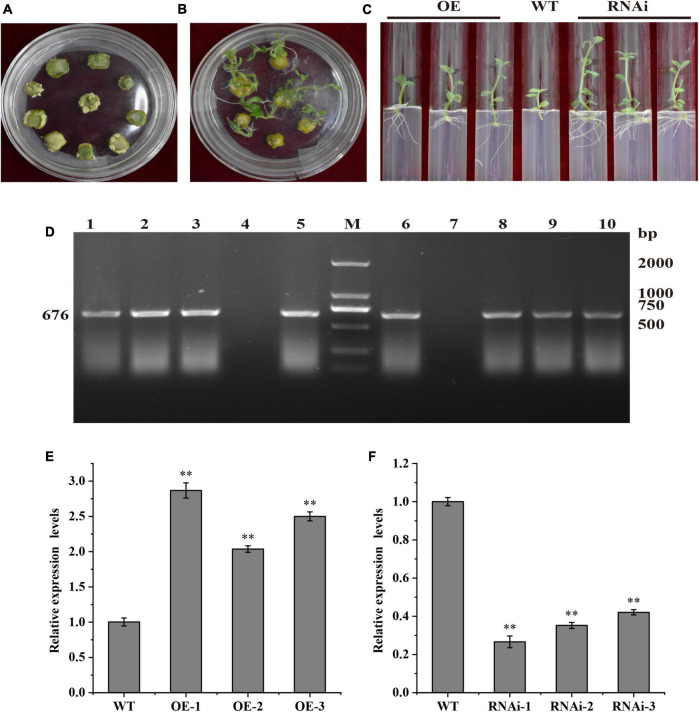
Acquisition and identification of transgenic potato. **(A)** Calli; **(B)** Differentiation and rooting transgenic plants; **(C)** Rooting and screening transgenic plants. OE, Transgenic plant “Desiree” carrying recombinant plasmids pBI121-StTCP15; WT, Wild-type plant “Desiree”; RNAi: Transgenic plant “Desiree” carrying recombinant plasmids pCPB121-amiR-StTCP15. **(D)** PCR detection of transgenic plants. M: DL 2000 marker; 1–3: pBI121-StTCP15 transformed plants; 4: Negative control; 5: Positive control pBI121-StTCP15 plasmid; 6: Positive control pCPB121-amiR-StTCP15 plasmid; 7: Negative control; 8–10: pCPB121-amiR-StTCP15 transformed plants. **(E,F)** The relative expression level *StTCP15* in the transgenic plants and WT plants. WT: Wild-type tubers of “Desiree”; OE-1∼OE-3: Transgenic tubers of “Desiree” carrying recombinant plasmids pBI121-StTCP15; RNAi-1∼RNAi-3: Transgenic tubers of “Desiree” carrying recombinant plasmids pCPB121-amiR-StTCP15. Each column represents the mean values ± SE (*n* = 3; *P < 0.05; **P < 0.01).

The potato cultivar “Desiree” plantlets *in vitro* were infected with *Agrobacterium tumefaciens* containing pBI121-StTCP15 overexpression vector and pCPB121-amiR-StTCP15 down-regulated expression vector, respectively, and were cultured in differentiation media to form calli and plants (Figure 3A). Transgenic plants were screened by rooting media containing kanamycin (Figures 3B,C).

The putative transformed buds were verified by amplification of reporter *NPT* II on the expression vector. Genomic DNA of transformed plants was extracted by CTAB method and analyzed by PCR and agarose gel electrophoresis. *NPT* II gene of 676 bp could be amplified from transgenic plants, but *NPT* II gene could not be amplified from WT plants, which was consistent with the expected experimental purpose. Therefore, this indicated that transgenic lines of overexpression pBI121-StTCP15 and down-regulated overexpression pCPB121-amiR-StTCP15 have been successfully obtained (Figure 3D).

### StTCP15 promotes sprouting of potato tubers

The total RNA of WT and transgenic plants were extracted and reverse transcribed into cDNA. The relative expression levels of *StTCP15* gene in dormancy released tubers were analyzed by qRT-PCR. The results showed that the relative expression level of *StTCP15* gene in pBI121-StTCP15 transgenic dormancy released tubers was significantly higher than that of WT, and the relative expression levels of OE-1, OE-2, and OE-3 were 2.87, 1.88, and 2.39 times of WT, respectively (Figure 3E). The relative expression of *StTCP15* gene in pCPB121-amiR-StTCP15 dormancy release tuber was significantly lower than WT, and the relative expression levels of RNAi-1, RNAi-2, and RNAi-3 were 0.27, 0.42, and 0.44 times of WT, respectively (Figure 3F).

**FIGURE 4:**
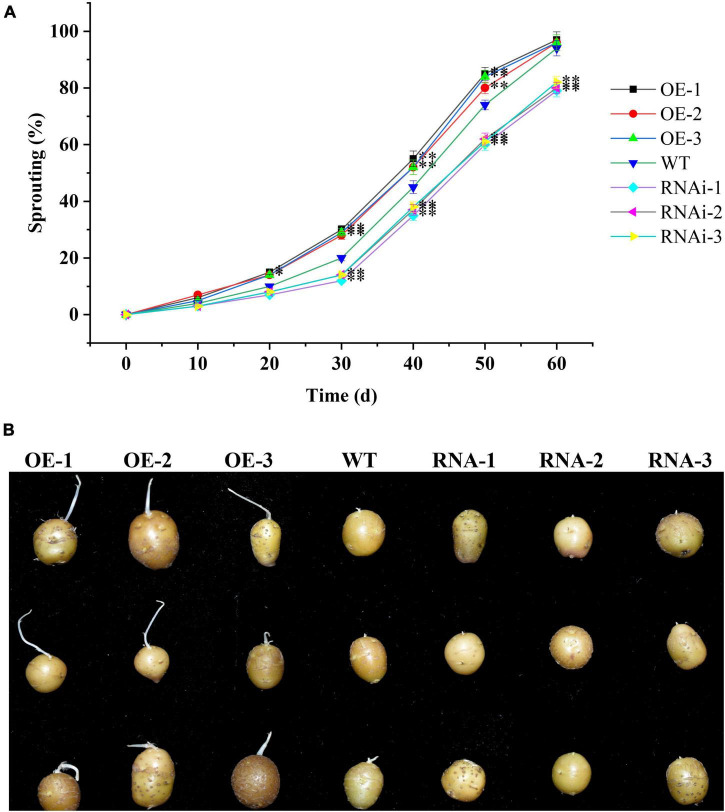
StTCP15 promotes sprouting of potato tubers. **(A)** Sprouting percentages of *StTCP15* transgenic and WT tubers. **(B)** Sprouting of transgenic tubers (partial) in 40 days. WT, Wild-type tubers of “Desiree”; OE-1∼OE-3, Transgenic tubers of “Desiree” carrying recombinant plasmids pBI121-StTCP15; RNAi-1∼RNAi-3, Transgenic tubers of “Desiree” carrying recombinant plasmids pCPB121-amiR-StTCP15.

To further clarify the role of *StTCP15* in potato dormancy release and sprouting, the dormancy release and sprouting of transgenic tubers were measured. According to Ittersum et al. (1992), when the sprouting percentage reached 80% and the length of at least one bud on the tuber was more than 0.2 cm as tubers sprouting time, the time of overexpression transgenic tubers (OE-1, OE-2, and OE-3) was 4.30, 3.67, and 3.54 d earlier than that of the wild type, respectively. However, the time for down-regulated expression transgenic tubers (RNAi-1, RNAi-2, and RNAi-3) was delayed by 6.16, 5.57, and 5.10 d, respectively, compared with WT (Figures 4A,B). These results showed that *StTCP15* could promote potato tuber dormancy release and sprouting.

### Analysis of superoxide dismutase, peroxidase, catalase, ABA, and GA_3_ in transgenic potato tuber

**FIGURE 5:**
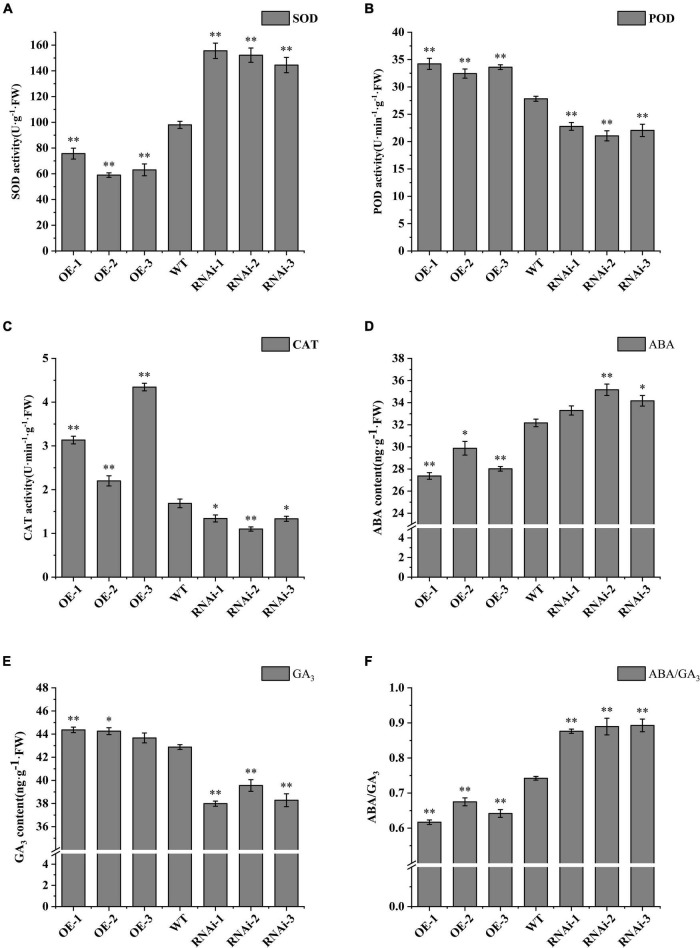
Antioxidant-related enzyme activity and endogenous hormone ABA/GA3 in tubers released from dormancy. **(A)** SOD activity; **(B)** POD activity; **(C)** CAT activity; **(D)** ABA content; **(E)** GA3 content; **(F)** ABA/GA3 ratio. WT: Wild-type tubers of “Desiree”; OE-1 ∼OE-4, Transgenic tubers of “Desiree” carrying recombinant plasmids pBI121-StTCP15; RNAi-1 RNAi-4, Transgenic tubers of “Desiree” carrying recombinant plasmids pCPB121-amiR-StTCP15. Each column represents the mean values SE (*n* = 3; *P < 0.05; **P < 0.01).

SOD, POD, and CAT, as protective enzymes of plant system defense system, can remove excessive free radicals and peroxides from potato tuber, increase the sugar content of potato tubers, and could help germination of plants (Gong et al., 2021). SOD activity of dormancy released tubers of overexpression (OE-1, OE-2, and OE-3) transgenic lines decreased by 22.8, 39.8, and 35.7%, respectively, compared to wild type, whereas SOD activity of down-regulated expression (RNAi-1, RNAi-2, and RNAi-3) transgenic lines increased by 58.7, 55.2, and 47.4%, respectively, compared to wild type (Figure 5A). POD activity increased by 23.0, 16.6, and 20.8% in overexpression transgenic lines, and decreased by 18.2, 24.4, and 20.6% in RNAi-n transgenic lines (Figure 5B). CAT activity increased significantly in overexpression transgenic lines compared with wild type, but decreased significantly in down-regulated expression transgenic lines (Figure 5C).

Abscisic acid and GA_3_ are the main endogenous hormones that antagonize seed dormancy and germination (Gubler et al., 2005; Finkelstein et al., 2008). The content of endogenous hormones ABA and GA_3_ was determined. The results showed that the ABA content of dormancy released tubers of OE-2 transgenic lines was significantly higher than that of wild type (*P* < 0.05), and the content of OE-1 and OE-3 transgenic lines was extremely significant (*P* < 0.01). Only OE-3 transgenic lines showed no difference in GA_3_ content, while OE-1 and OE-2 transgenic lines showed extremely significant differences (*P* < 0.01) and significant differences (*P* < 0.05), respectively. There was no difference in ABA content of dormancy released tubers of RNAi-1 transgenic lines, but there was an extremely significant difference (*P* < 0.01) and significant difference (*P* < 0.05) in RNAi-2 and RNAi-3 transgenic lines. The content of GA_3_ in dormancy released tubers of down-regulated expression (RNAi-1, RNAi-2, and RNAi-3) transgenic lines were extremely significantly higher than that of the wild type (*P* < 0.01; Figures 5D,E). It has been shown that seed germination is not determined by ABA or GA_3_ alone, but by the dynamic balance of endogenous ABA and GA_3_ levels in seeds (Seo et al., 2009; Liu and Hou, 2018; Chen et al., 2020). Therefore, the ratios of ABA and GA_3_ in dormancy released tubers was compared in this study. The results showed that the ABA/GA_3_ ratio in OE-1, OE-2, and OE-3 transgenic lines were 0.62, 0.67, and 0.64, respectively, which was extremely significantly lower than that in the wild type (0.74). The ABA/GA_3_ ratio of RNAi-1, RNAi-2, and RNAi-3 transgenic lines were 0.88, 0.89, and 0.87, respectively, which were extremely significantly higher than those of the wild type (Figure 5F). According to the dynamic equilibrium theory, a high ABA/GA_3_ ratio is beneficial to dormancy, while a low ABA/GA_3_ ratio is beneficial to germination, suggesting that *StTCP15* has the function of positively regulating dormancy release of potato tubers.

### StTCP15 interacts with StSnRK1, StF-box and StGID1

**FIGURE 6:**
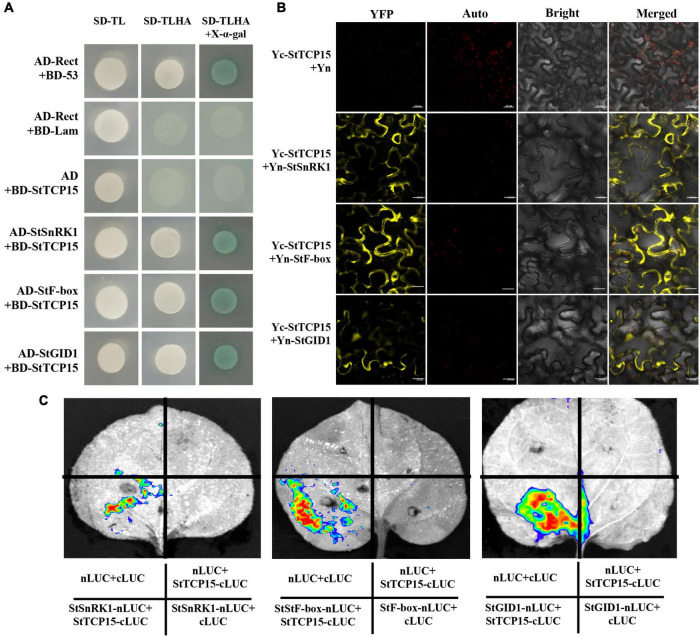
**(A)** The Y2H reciprocal interactions between the interacting protein and the StTCP15 protein. AD, pGADT7. SD-TL stands for -Trp-Leu auxotrophic media; SD-TLHA stands for -Trp-Leu-His-Ade auxotrophic media, where x-α-gal was added to promote blue. **(B)** BiFC analysis was used to detect the interaction of StTCP15 in tobacco. Yc-StCTP15 + Yn-StSnRK1/StF-bos/StGID1 was used as the experimental group; Yc-StCTP15 + Yn were used as negative controls; Yellow indicates YFP fluorescence. The scale bale represents 20 μm. **(C)** Split-LUC complementation assay showing that StTCP15 could interact with StSnRK1, StF-box and StGID1 in cells of *Nicotiana benthamiana* leaves, respectively. The LUC signals were not detected in the corresponding negative controls.

To further explore the molecular mechanism of StTCP15 in regulating potato tuber sprouting, yeast two-hybrid screening was performed to identify the proteins interacting with StTCP15. Among the various proteins identified, we focused on StSnRK1 (XM_006345112.2), StF-box (CP046683.1) and StGID1 (XM_006358383.1) for further analysis, because they were previously reported to affect seed germination (McGinnis et al., 2003; Qu et al., 2020; Song et al., 2021). Yeast cells co-transformed by pGADT7-StSnRK1, pGADT7-StF-box and pGADT7-StGID1 with pGBKT7-StTCP15 and cells transformed by positive control plasmid were grown on SD/-Ade/-His/-Leu/-Trp/X-α-gal media, respectively. Moreover, it turned blue on SD/-Ade/-His/-Leu/-Trp/X-α-gal media, respectively, while the negative control did not (Figure 6A). This demonstrated that StTCP15 interacted with StSnRK1, StF-box and StGID1, respectively. To further characterize this interaction, BiFC experiments were performed. It is connected StTCP15 to pSPYCE-35S, the interacting protein to pSPYNE-35S, and mixed injection into the *N. benthamiana* leaves. YFP fluorescence signal was found to be expressed in both the nucleus and the cell membrane (Figure 6B). This is consistent with the subcellular localization of StTCP15 in the nucleus and cell membrane (Figure 2). In addition, Split-LUC Complementation analysis of *N. benthamiana* leaves showed that only LUC signals were detected in the combined region of StSnRK1, StF-box and StGID1 by StTCP15, and no LUC signals were detected in other regions (Figure 6C). Based on these results, this research concluded that StTCP15 interacts with StSnRK1, StF-box and StGID1, respectively.

## Discussion

In the whole life cycle of the potato tuber, tuber dormancy and sprouting are the key components of tuber physiological characteristics, which determine the growth and yield of the potato (Sonnewald and Sonnewald, 2014). The dormancy release process of potato tubers was the result of a variety of molecular regulations working together. However, the participation of specific molecular mechanisms in potato dormancy release remains to be seen (Gong et al., 2021).

T are plant-specific transcription factors involved in multiple processes of biological function (Broholm et al., 2008; Tatematsu et al., 2008; Koyama et al., 2010; Danisman et al., 2012; Davière et al., 2014; Nicolas et al., 2015; Wu et al., 2019). In the previous study, Wang et al. (2019) found that the expression level of PCF gene *StTCP15* was continuously up-regulated during the dormancy release of potato tubers. In this research, the relative expression level of *StTCP15* was analyzed during the tuber dormancy release of potato cultivar “Desiree” by qRT-PCR (Figure 1C) and it was steady with the results of Wang et al. (2019). By constructing PCF class and StTCP15 homologous evolutionary tree of *A. thaliana*, it was found that StTCP15 sequence was related to AtTCP14/15 (Figure 1A). Steiner et al. (2012) showed that AtTCP14 and AtTCP15 regulate cell proliferation in developing leaves and specific flower tissues. Tatematsu et al. (2008) showed a functional relationship between *AtTCP14* and GA, and the activation of *AtTCP14* used to be vital for seed germination. Lacking this activity, seeds are hypersensitive to the negative effects on germination of GA biosynthesis inhibitor paclobutrazol. Resentini et al. (2021) research showed that *AtTCP14* and *AtTCP15* mediate the promotion of seed germination by gibberellins in *A. thaliana*. In this study, the *StTCP15* gene was transformed into potato cultivar “Desiree” by *Agrobacterium*-mediated method. Phenotypic analysis confirmed that *StTCP15* gene had no effect on potato plants (Supplementary Figure 3). Overexpression potato tubers dormancy released and sprouted earlier than wild-type tubers, while down-regulated expression tubers delayed dormancy released and sprouted (Figures 4A,B). The results showed that *StTCP15* gene promotes the dormancy release and sprouting of potato tubers.

The activity of antioxidant enzymes and the content of endogenous hormones ABA and GA_3_ in transgenic tubers had been determined. It was found that SOD activity decreased, and POD activities and CAT activities increased in dormancy released tubers of overexpression transgenic lines (Figures 5A–C), which was the same as results of Wang et al. (2020). Gong et al. (2021) showed that POD and CAT can remove excessive free radicals and peroxides in plants, increase the sugar content in potato tubers and promote sprouting. In overexpression transgenic lines, ABA content was always significantly lower than that of wild type and inhibited transgenic lines, and GA_3_ was always significantly higher than that of wild type and inhibited transgenic lines (Figures 5D,E). However, studies have shown that seed germination is not affected by ABA or GA_3_ hormones, and is determined by the dynamic balance of endogenous ABA and GA_3_ levels in seeds. A low ABA/GA_3_ ratio is conducive to seed dormancy release and germination (Finkelstein et al., 2008; Seo et al., 2009; Yang et al., 2020). In the research, ABA/GA_3_ ratio in overexpression transgenic lines was extremely significantly lower than that in the wild type, while ABA/GA_3_ ratio in down-regulated expression transgenic lines was extremely significantly higher than that in the wild type (Figure 5F). Teh et al. (2014) showed that during the ripening process of oil palm fruits, the increase in ABA/GA_3_ ratio was consistent with ripening. Ge (2019) found that GA_3_/ABA ratio of oat seeds with different maturity increased with storage time, and ABA/GA_3_ ratio was positively correlated with germination rate of oat seeds. Ling and Zhang (2022) recently reported that ABA levels were significantly reduced during seed development in *Paris polyphylla* var. *yunnanensis*, while GA_3_ levels were significantly increased in nine endogenous gibberellins (GAs), resulting in a significantly increased GA_3_/ABA ratio in germinated seeds. These results provide evidence that *StTCP15* regulates the dynamic balance of endogenous ABA and GA_3_ levels during the dormancy release and sprouting of potato tubers.

**FIGURE 7:**
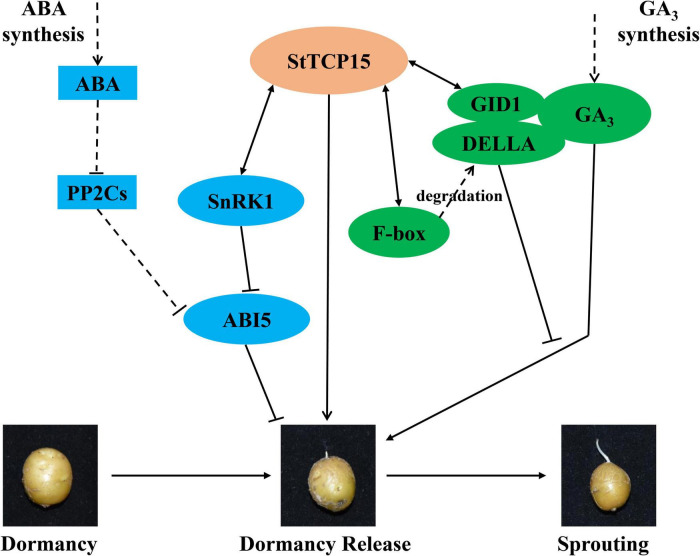
The putative pathway model of StTCP15 in potato tuber dormancy release. StTCP15 regulates the dormancy and sprouting of potato tubers by regulating the dynamic balance between ABA and GA_3_. GID1 senses GA signals promotes the formation of GID1/GA/DELLA complex, and SLY1 F-Box protein recognizes, binds and degrades DELLA, thus promoting the dormancy release and sprouting of tubers. SnRK1 binds to ABI5, down-regulates the expression of ABI5, participates in ABA signal transduction, and regulates tuber dormancy. Solid lines represent pathways that have been experimentally confirmed to have direct or inhibitory effects, and dashed lines represent pathways that have indirect stimulatory and inhibitory effects.

In order to further clarify the molecular mechanism of StTCP15 in the dormancy release and sprouting of potato tubers, the proteins interacting with StTCP15 were screened using Y2H system (Figure 6A), which were as follows: StSnRK1, StF-box and StGID1 have been proved by BiFC and Split-LUC techniques (Figures 6B,C). Resentini et al. (2021) found that AtTCP14 and AtTCP15 interact with DELLA protein GAI and RGL2 to mediate GA to promote *A. thaliana* seed germination. It has also been reported that AtTCP14, through its interaction with DOF6, indirectly inhibits ABA biosynthesis gene ABA1 and ABA-regulated related stress genes, resulting in delayed germination (Rueda-Romero et al., 2012). Song et al. (2021) found that SnRK1 binds to ABI5 and down-regulates the expression of ABI5, thereby affecting ABA signal transduction and promoting seed germination. F-box protein is the most important SCF (SKP1, Cullin/CDC53, F-box protein) complex subunit in Ubiquitin (Ub)-26S proteasome system. It mediates various physiological processes from hormone signaling cascade to environmental stress response (Santner and Estelle, 2010). Song et al. (2012) found that the overexpression of *OsFbx352* reduced the expression of genes involved in ABA synthesis and increased the expression of genes encoding ABA catabolism, and the germination of seeds overexpressing *OsFbx352* was less inhibited by glucose than that of non-transgenic seeds. GID1, as GA receptor, promotes the formation of GID1/GA/DELLA complex, leading to DELLA degradation by 26S proteasome and release of transcription factors (Ariizumi et al., 2011), thus releasing seed dormancy in advance. Combined with the experimental results, it was proposed the pathway model of StTCP15 regulating potato tuber dormancy and sprouting (Figure 4C), and StTCP15 may interact with StSnRK1 to regulate the expression of ABI5, and thus regulate the ABA signaling pathway. StTCP15 interacts with StF-Box and StGID1 to reduce DELLA expression and regulate GA_3_ signaling (Figure 7). However, it is still not clear whether StSnRK1, StF-box and StGID1 proteins are necessary for regulating potato tuber dormancy and sprouting, and further experiments are needed to prove it.

## Conclusion

In this study, the potato *StTCP15* gene was cloned. qRT-PCR analysis showed that *StTCP15* gene was expressed in different tissues of potato, the relative expression level in bud was higher than that in tuber, and the relative expression level continued to increase during tuber dormancy release. The expression of *StTCP15* gene reduced SOD activity, increased POD activity and CAT activity in the antioxidant system of potato tubers. It was found that *StTCP15* could promote sprouting of potato tubers by affecting the dynamic balance between ABA and GA. These results could provide insights into the molecular mechanisms of potato tuber dormancy and sprouting and their potential roles in genetic improvement.

## Data availability statement

The datasets presented in this study can be found in online repositories. The names of the repository/repositories and accession number(s) can be found in the article/Supplementary material.

## Author contributions

HS and NZ conceived and designed the experiments. KW, XF, HZ, SL, XP, and XW performed the laboratory experiments. KW, NZ, and HS performed the data analysis and interpretation and wrote the manuscript. All authors contributed to the article and approved the submitted version.

## Footnotes

1
http://spuddb.uga.edu

2
http://web.expasy.org/protparam/

3
https://web.expasy.org/cgi-bin/protscale/protscale.pl/

4
http://www.cbs.dtu.dk/services/NetPhos/

5
https://www.takarabio.com/learning-centers/cloning/primer-design-and-other-tools
