# Supplementary material for: StTCP15 regulates potato tuber sprouting by modulating the dynamic balance between abscisic acid and gibberellic acid
Source: Front Plant Sci. 2022 Sep 16;13:1009552. doi: 10.3389/fpls.2022.1009552 (PMC9523429; doi:10.3389/fpls.2022.1009552)
Supplement: Supplementary file 1 [file Data_Sheet_1.docx]

# Additional files


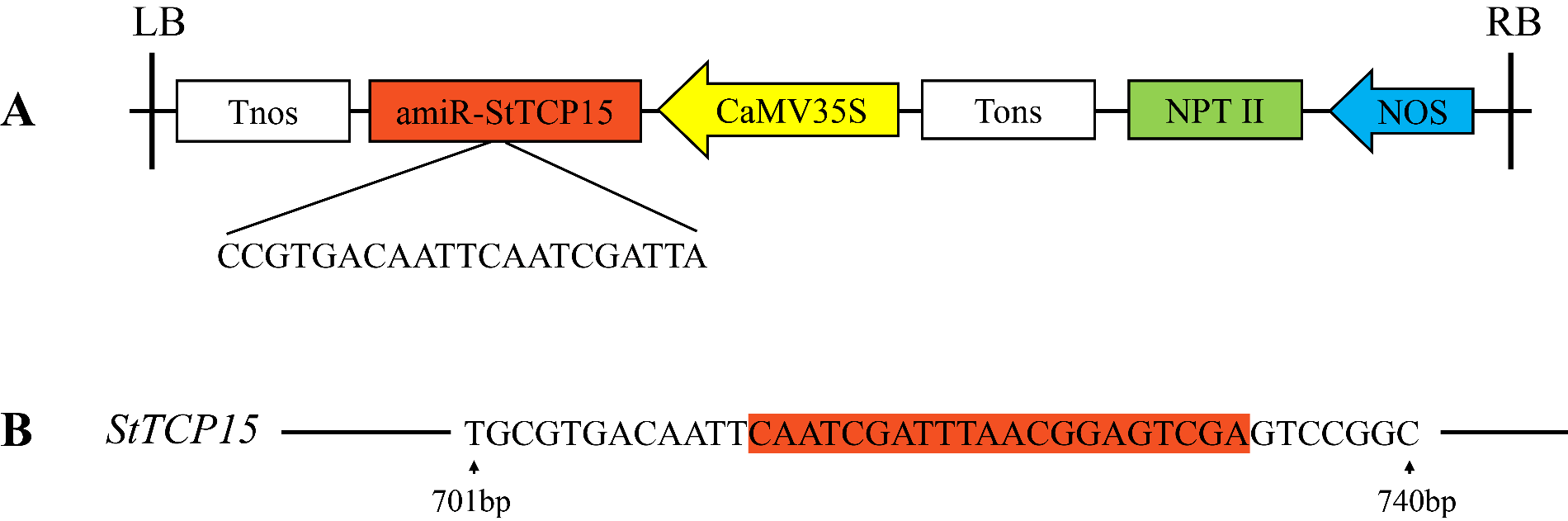


**Supplementary Figure 1** amiRNA-mediated gene silence in transgenic potato plants. **(A)** Schematic illustration of the engineered pCPB121-amiR-StTCP15 vector. **(B)** Schematic illustration of the target region of *StTCP15* gene.


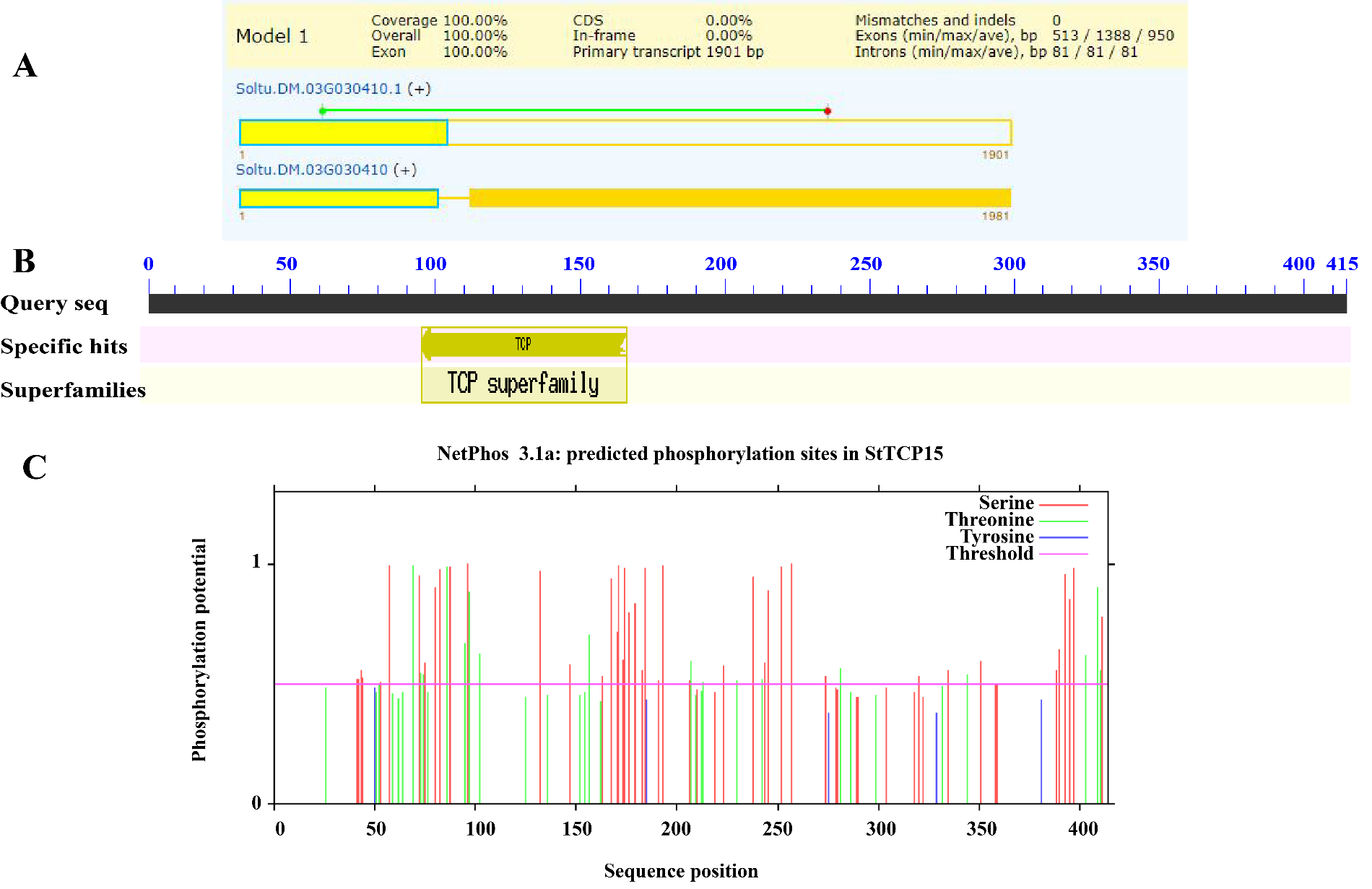


**Supplementary Figure 2**: Bioinformatics analysis of StTCP15. **(A)** Exon-intron structure of *STCP15*, **(B)** Schematic diagram of the domains structures of StTCP15 protein. **(C)** Phosphorylation site of STCP15 protein.


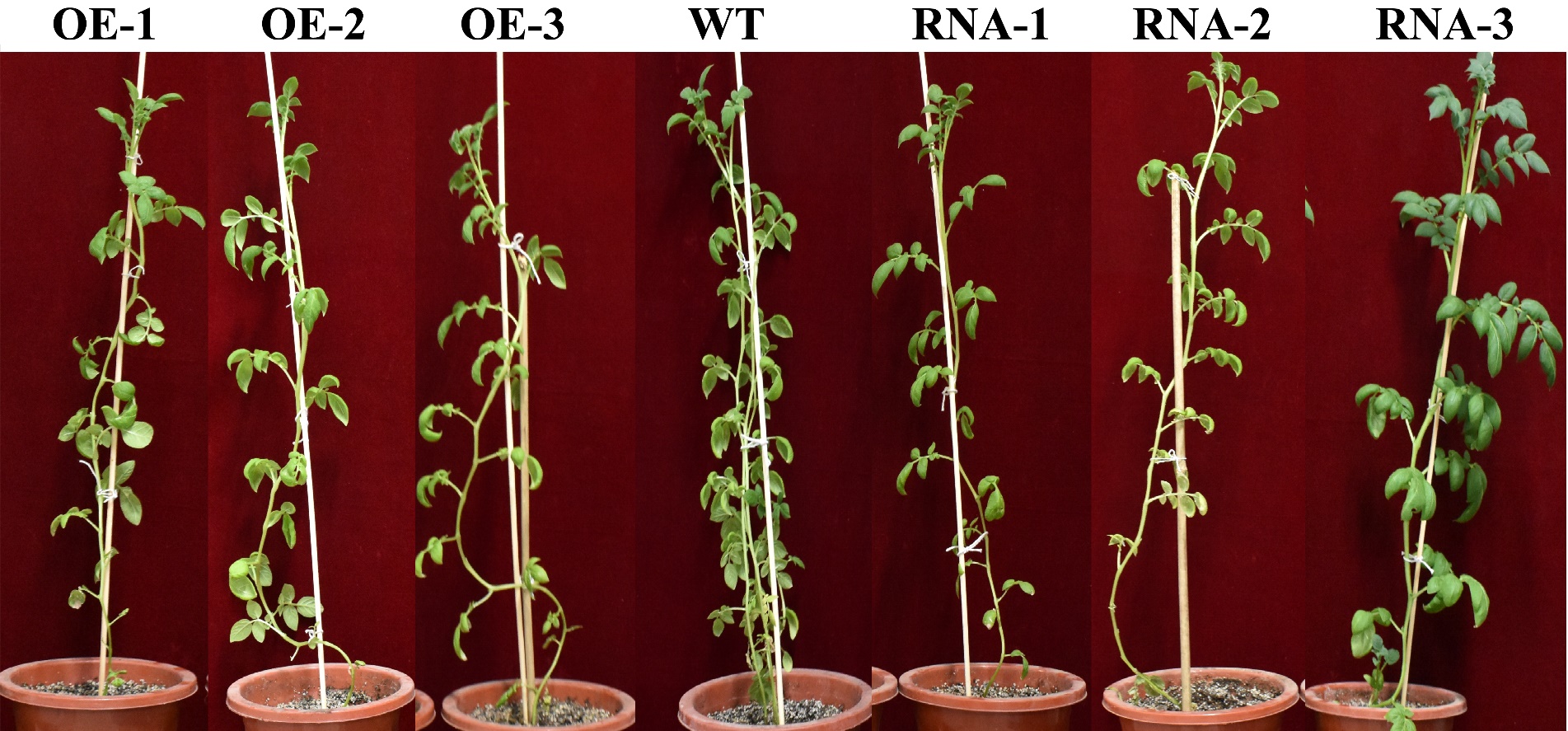


**Supplementary Figure 3:** Phenotypic identification of transgenic potato. WT: Wild-type tuber “Desiree”; OE-1~OE-3: Transgenic tuber “Desiree” carrying recombinant plasmids pBI121-StTCP15; RNAi-1~RNAi-3: Transgenic tuber “Desiree” carrying recombinant plasmids pCPB121-amiR-StTCP15;
