# Supplementary material for: StTCP15 regulates potato tuber sprouting by modulating the dynamic balance between abscisic acid and gibberellic acid
Source: Front Plant Sci. 2022 Sep 16;13:1009552. doi: 10.3389/fpls.2022.1009552 (PMC9523429; doi:10.3389/fpls.2022.1009552)
Supplement: Supplementary file 2 [file Table_2.DOCX]

**Supplementary Table 1** Specific Primers used for PCR and qRT-PCR.

| PCR primers for the recombinant plasmid | | |
| --- | --- | --- |
| pCAMBIA1300-EGFP-StTCP15 | Forward | 5′-ACGGGGGACGAGCTCGGTACCATGGATGGAGGAGGTGATGATC-3′ |
|  | Reverse | 5′-GCTCACCATGTCGACTCTAGATGAATGATGGCTCGTTGTATCG-3′ |
| pBI121-StTCP15 | Forward | 5′-ACGGGGGACTCTAGAGGATCCATGGATGGAGGAGGTGATGATC-3′ |
|  | Reverse | 5′-CGATCGGGGAAATTCGAGCTCCTATGAATGATGGCTCGTTGTATCG-3′ |
| pCPB121-aimRNA-StTCP15 | amiRNA | 5′-TAATCGATTGAATTGTCACGG-3′ |
|  | I miR-s | 5′-GATAATCGATTGAATTGTCACGGTCTCTCTTTTGTATTCC-3′ |
|  | II miR-a | 5′-GACCGTGACAATTCAATCGATTATCAAAGAGAATCAATGA-3′ |
|  | III miR*s | 5′-GACCATGACAATTCATTCGATTTTCACAGGTCGTGATATG-3′ |
|  | IV miR*a | 5′-GAAAATCGAATGAATTGTCATGGTCTACATATATATTCCT-3′ |
|  | A | 5′-CTGCAAGGCGATTAAGTTGGGTAAC-3′ |
|  | B | 5′-GCGGATAACAATTTCACACAGGAAACAG-3′ |
| pGBKT7-StTCP15 | Forward | 5′-TCAGAGGAGGACCTGCATATGATGGATGGAGGAGGTGATGATC-3′ |
|  | Reverse | 5′-GGTTATGCTAGTTATGCGGCCGCCTATGAATGATGGCTCGTTGTATCG-3′ |
| pGADT7-StSnRK1 | Forward | 5′-CAGATTACGCTCATATGATGGTGGTTCATCGGGACC-3′ |
|  | Reverse | 5′-CGAGCTCGATGGATCCTCATTGTGGTCCCTCTAGCTGC-3′ |
| pGADT7-StF-box | Forward | 5′-CAGATTACGCTCATATGATGAATCAAAATGAGTTCACTGAA-3′ |
|  | Reverse | 5′-CGAGCTCGATGGATCCTCAGATCTCCACACAGCATCCA-3′ |
| pGADT7-StGID1 | Forward | 5′-CAGATTACGCTCATATGATGGTGGACACTAAAGAGATC-3′ |
|  | Reverse | 5′-CGAGCTCGATGGATCCCTAGGAATGGTTAGGATGTATAAAG-3′ |
| pSPYCE-StTCP15 | Forward | 5′-CGCCACTAGTGGATCCATGGATGGAGGAGGTGATGATC-3′ |
|  | Reverse | 5′-GTATGGGTACATCCCGGGTGAATGATGGCTCGTTGTATCGTG-3′ |
| pSPYNE-StSnRK1 | Forward | 5′-CGCCACTAGTGGATCCATGGTGGTTCATCGGGACC-3′ |
|  | Reverse | 5′-CTTTTGCTCCATCCCGGGTTGTGGTCCCTCTAGCTGCC-3′ |
| pSPYNE-StF-box | Forward | 5′-CGCCACTAGTGGATCCATGAATCAAAATGAGTTCACTGAA-3′ |
|  | Reverse | 5′-CTTTTGCTCCATCCCGGGGATCTCCACACAGCATCCAGA-3′ |
| pSPYNE-StGID1 | Forward | 5′-CGCCACTAGTGGATCCATGGTGGACACTAAAGAGATCAACA-3′ |
|  | Reverse | 5′-CTTTTGCTCCATCCCGGGGGAATGGTTAGGATGTATAAAGCT-3′ |
| pCAMBIA1300-cLUC- StTCP15 | Forward | 5′-ACGCGTCCCGGGGCGGTACCATGGATGGAGGAGGTGATG-3′ |
|  | Reverse | 5′-CGCCGGGCCCTCTAGACTATGAATGATGGCTCGTTGTATCG-3′ |
| pCAMBIA1300-nLUC-StSnRK1 | Forward | 5′-AGAACACGGGGGACGAGCTCATGGTGGTTCATCGGGACC-3′ |
|  | Reverse | 5′-ACGAGATCTGGTCGACTTGTGGTCCCTCTAGCTGCC-3′ |
| pCAMBIA1300-nLUC-StF-box | Forward | 5′-AGAACACGGGGGACGAGCTCATGAATCAAAATGAGTTCACTGAA-3′ |
|  | Reverse | 5′-ACGAGATCTGGTCGACGATCTCCACACAGCATCCAGA-3′ |
| pCAMBIA1300-nLUC-StGID1 | Forward | 5′-AGAACACGGGGGACGAGCTCATGGTGGACACTAAAGAGATCAACA-3′ |
|  | Reverse | 5′-ACGAGATCTGGTCGACGGAATGGTTAGGATGTATAAAGCT-3′ |
| **qRT-PCR primers for the plant** | | |
| StTCP15 | Forward | 5′-TCCACCAAAGACCGCCATAC-3′ |
|  | Reverse | 5′-GGTTCCTGTAGCGGCGATTA-3′ |
| StEf1α | Forward | 5′-CAAGGATGACCCAGCCAAG-3′ |
|  | Reverse | 5′-TTCCTTACCTGAACGCCTGT-3′ |
| **PCR primers for the transgenic plant** | | |
| NPTII | Forward | 5′-GCTATGACTGGGCACAACAG-3′ |
|  | Reverse | 5′-ATACCGTAAAGCACGAGGAA-3′ |
